# Impact of time in range during hospitalization on clinical outcomes in diabetic patients with toe amputation: a propensity score matching analysis

**DOI:** 10.1186/s12893-022-01762-1

**Published:** 2022-08-12

**Authors:** Su Li, Ze-Xin Huang, Dong-hao Lou, Ye-Yong Jiang, Sheng Zhao

**Affiliations:** 1 Burn and Wound Healing Center, The First Affiliated Hospital of Wenzhou Medical University, Shangcai Village, Wenzhou, 325000 Zhejiang China; 2 Department of Urinary Surgery, Taizhou First People’s Hospital, Taizhou, China; 3 Department of Hepatobiliary Surgery, The First Affiliated Hospital of Wenzhou Medical University, Wenzhou, China; 4 Department of Urinary Surgery, Taizhou First People’s Hospital, Taizhou, China

**Keywords:** Amputation, Diabetic foot, HbA1c, Re-amputation, Time in rang

## Abstract

**Purpose:**

In recent years, time in range (TIR), defined as a percentage within a target time range, has attracted much attention. This study was aimed to investigate the short-term effects of Time in Rang on diabetic patients undergoing toe amputation in a more specific and complete manner.

**Methods:**

A retrospective analysis on patients with diabetic foot ulcer (DFU) treated by toe amputation or foot amputation at the First Affiliated Hospital of Wenzhou Medical University between January 2015 and December 2019 were evaluated. A 1:1 match was conducted between the TIR < 70% group and the TIR ≥ 70% group using the nearest neighbor matching algorithm. Data were analyzed using Chi-squared, Fisher’s exact, and Mann–Whitney U tests.

**Results:**

Compared with patients in the TIR ≥ 70% group, patients in the TIR < 70% had a higher rate of re-amputation, and a higher rate of postoperative infection. Multivariate analysis revealed that smoking, lower extremity arterial disease and TIR < 70% were risk factors for surgery of re-amputation. The results of subgroup analysis found that the TIR < 70% was associated with a greater risk of re-amputation in patients with HbA1c < 7.5%, lower extremity arterial disease, and non-smokers.

**Conclusions:**

TIR can be used as a short-term glycemic control indicator in patients with DFUs and should be widely accepted in clinical practice. However, a future multicenter prospective study is needed to determine the relationship between TIR and toe re-amputation in diabetic foot patients.

## Introduction

Recently, the increasing incidence of diabetes has resulted in the occurrence of related complications, thus reducing people’s quality of life [1]. Diabetic foot ulcer (DFU) is one of the most dangerous and common complications in elderly patients with diabetes mellitus, often resulting in forced toe amputation [2, 3]. It is well known that DFU is caused by the interaction of multiple factors caused by persistent, uncontrolled hyperglycemia, accompanied by infection, ulceration and deep tissue destruction [4]. However, poor blood sugar control can lead to life-threatening complications such as fungal, foot damage, cardiovascular disease and bacterial infections, and memory deficits [5]. In addition, the 5-year mortality rate of patients with diabetes who have previously experienced amputation is more than half, which is of great concern to us [6, 7]. In other words, diabetic patients who have undergone toe amputation need to be paid more attention, because the risk of re-amputation tends to be higher. Therefore, if the occurrence of toe amputation in patients with DFU can be avoided as much as possible, the quality of life and survival rate of patients will be greatly improved. A further prediction of amputation risk and appropriate management of risk factors could lead to improved cure rates, lower amputation rates and lower treatment costs for early treatment [8].

A meta-analysis shows that stabilizing blood glucose (HbA1c as a marker of blood glucose control) has the ability to significantly reduce the risk of amputation in patients with diabetic foot ulcers [9]. This finding demonstrates that stable blood glucose control plays an essential role in preventing complications in patients with diabetes, especially DFUs. Mounting evidence shown that hemoglobin A1c (HbA1c) is an important indicator of blood glucose control in diabetic patients in the past 2–3 months or so, and is often used as the gold standard [10, 11]. However, HbA1c has some limitations because it does not provide information about daily short-term fluctuations in blood glucose. Further studies showed that there was a considerable difference between HbA1c and daily mean blood glucose, even in the same patient [12]. Therefore, it is urgent to find an index that can evaluate short-term blood glucose fluctuation to make up for the deficiency of HbA1c.

With the advent of continuous glucose monitoring (CGM), the time in range (TIR) derived from CGM data may be a valuable indicator for assessing glucose control [13]. In recent years, TIR, defined as a percentage within a target time range (blood glucose is typically 3.9–10.0 mmol/L), has attracted much attention. The effectiveness of using the seven-point test and the correlation of TIR with the risk of diabetic retinopathy and diabetic nephropathy have been demonstrated [14]. Many studies have indeed reported risk factors for amputation in DFU patients [15–17]. Compared to HbA1c, the TIR appears to have an impact on short-term outcomes in patients with diabetic foot as an indicator of short-term glucose fluctuations. Unfortunately, few studies have reported the effects of TIR on diabetic patients with diabetic toe amputation. This study was aimed to investigate the short-term effects of Time in Rang on diabetic patients undergoing toe amputation in a more specific and complete manner.

## Methods

### Study population

The research protocol was approved by the ethics committee of the First Affiliated Hospital of Wenzhou Medical University (No.2020116). Data from patients with diabetic foot ulcer (DFU) treated by toe amputation or foot amputation at the First Affiliated Hospital of Wenzhou Medical University between January 2015 and December 2019 were evaluated. The inclusion criteria were: (a) patients were diagnosed with diabetic foot ulcer, (b) patients underwent toe amputation, and (c) patients were followed up in the outpatient department for 1 year. The exclusion criteria were: (a) patients were diagnosed with other short-term life-threatening conditions, such as cancer, (b) patients who underwent foot amputation or major amputation, and (c) patients with incomplete or inaccurate medical records. All patients were followed up half a month after amputation. All patients were followed up for 1 year by telephone or outpatient follow-up every 2–3 months thereafter. The study was conducted according to the principles of the Declaration of Helsinki.

### Data collection

Data on eligible patients from the medical system were obtained and retrospectively studied. The following information was collected: (1) characteristics at admission, including age, gender, smoking history, drinking history, and body mass index (BMI); (2) preoperative blood index, including blood glucose, HbA1c, WBC, neutrophil, lymphocyte, platelet, CRP, anemia, albumin, creatinine; (3) disease-related information, including hypertension, kidney disease, lower extremity arterial disease (LEAD), preoperative bacterial culture, Wagner score; (4) operative information, including number of toes amputated; and (5) clinical outcome, including toe amputation again within six months, postoperative infection, length of stay (LOS), costs, and incision healing on discharge.

### Definition of time in range

Since CGM is not widely used in surgical wards, TIR is currently based on data from seven blood glucose tests. Here, blood samples were collected from each patient's daily seven-point blood glucose test to assess TIR throughout the hospital stay. Seven Point Glucose test was performed by a nurse who measured capillary blood glucose levels before, 2 h after each meal and at bedtime in patients with diabetic foot. In addition, previous studies have also shown that the glucose index calculated based on the seven -point glucose test is similar to that of CGM [18]. Although less widely used than the CGM, the seven-point glucose test is of great value when the percentage of missing values per patient is less than 30% and blood glucose measurements are more than ten days old during hospitalization. TIR values were represented by glucose levels of 3.9–10.0 mmol/L. Then the percentage of times of TIR and total times of test within the range was calculated to calculate the TIR value of each patient, as shown below: times of TIR/ total times of test × 100%. In previous studies, TIR ≥ 70% and TIR ≥ 50% have been used as a standard threshold [19, 20]. However, since the threshold of 70% is more common in related studies, 70% was selected as the indicator of poor blood glucose control in this study.

### Propensity score matching

Compared to prospective clinical studies, retrospective studies are more likely to show differences between groups. In order to reduce differences in baseline data of patients enrolled in the study, the propensity score matching (PSM) will be applied to the study group to obtain a more reliable comparison. A 1:1 match was conducted between the TIR < 70% group and the TIR ≥ 70% group using the nearest neighbor matching algorithm and a caliper of 0.2. In the logistic regression model of PSM, we included the factors considered to be associated with patients’ diabetes as covariables. The relevant factors included were blood glucose, HbA1c, leukocyte, neutrophil, albumin, and hypertension.

### Statistical analysis

Categorical variables were displayed as numbers (%) and a chi-square test or Fisher’s exact test was performed for them. According to the Kolmogorov–Smirnov test, all continuous variables in this study were divided into normal distribution and non-normal distribution and a student's t-test or Mann–Whitney U test was performed for them. Non-normally distributed data is expressed as the medians and interquartile ranges (IQRs), and normally distributed continuous variables were expressed as means ± standard deviation (SD). Univariate and multivariate logistic regression were performed to evaluate risk factors for postoperative complications and confirm the relationship between toe re-amputation and TIR using a method of entry. Subgroup analysis was performed using univariate logistic regression analysis. Statistical analyses were performed using SPSS software 22.0 (IBM Corp., Armonk, NY, USA), while PSM was conducted using R version 3.6.2. Analysis items with *P*-values < 0.05 represented a statistically significant, and all tests were 2-sided.

## Results

### Patient characteristics

**Fig. 1:**
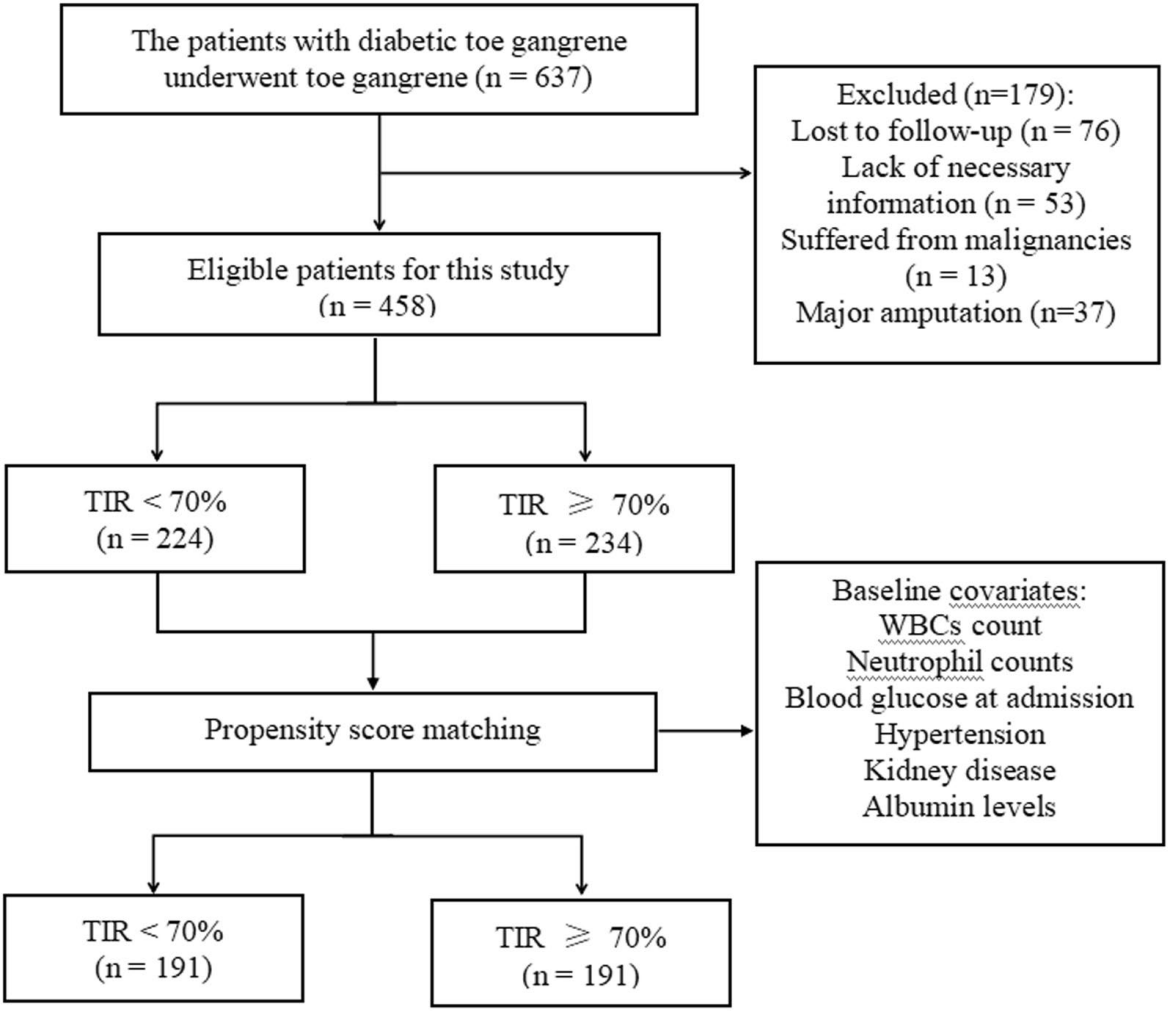
Population flowchart

In our study, a total of 637 patients with diabetic foot underwent toe amputation. Of these, 179 were excluded for one of the following reasons: (a) lost to follow-up, (b) lack of necessary information, (c) suffered from malignancies, and (d) major amputation. After considering exclusion criteria, a total of 458 patients were selected in our study. The patient flow chart for this study is shown in Fig. 1. Because of the difference in baseline data between the two groups, these patients received a 1:1 propensity-score match. Before matching, the TIR < 70% group had a higher WBCs count, higher neutrophil counts, higher blood glucose at admission, lower rates of hypertension, lower rates of kidney disease, and lower albumin levels. After matching, the baseline data of 191 patients in each group were balanced and there was no statistical difference. The characteristics of patients before and after propensity score matching were shown in Table 1.

**Table 1:** Clinical Characteristics of Patients Before and After Propensity Score Matching

Characteristic	Before propensity score matching			After propensity score matching		
	TIR < 70 (n = 224)	TIR ≥ 70 (n = 234)	*P*-value	TIR < 70 (n = 191)	TIR ≥ 70 (n = 191)	*P*-value
Age, median (IQR), years	67.0 (60.0–74.0)	67.0 (61.0–73.8)	0.937	67.0 (60.0–74.0)	67.0 (59.5–72.5)	0.670
Sex, n (%)						
Male	148 (66.1%)	157 (67.1%)	0.817	132 (69.1%)	130 (68.1%)	0.826
Female	76 (33.9%)	77 (32.9%)		59 (30.9%)	61 (31.9%)	
BMI, median (IQR), kg/m^2^	23.0 (20.9–25.0)	22.9 (21.3–25.4)	0.707	23.0 (21.0–25.0)	22.9 (21.3–25.4)	0.971
Preoperative blood glucose, n (%)						
< 6.1	35 (15.6%)	80 (34.2%)	< 0.001	35 (18.3%)	47 (24.6%)	0.135
≥ 6.1	189 (84.4%)	154 (65.8%)		156 (81.7%)	144 (75.4%)	
Preoperative HbA1c, n (%)						
< 7.5	46 (20.5%)	62 (26.5%)	0.133	43 (22.5%)	43 (22.5%)	1.000
≥ 7.5	178 (79.5%)	172 (73.5%)		148 (77.5%)	148 (77.5%)	
Preoperative WBC, median (IQR)	9.5 (7.1–12.6)	8.4 (6.5–11.6)	0.004	9.3 (7.0–12.2)	8.6 (6.7–11.8)	0.164
Preoperative neutrophil, median (IQR)	6.9 (5.1–10.2)	5.9 (4.2–8.8)	0.004	6.6 (4.9–9.8)	6.1 (4.4–9.0)	0.215
Preoperative lymphocyte, median (IQR)	1.5 (1.1–1.8)	1.5 (1.1–1.9)	0.420	1.4 (1.1–1.8)	1.5 (1.1–1.8)	0.769
Preoperative platelet, median (IQR)	267.0 (215.0–354.2)	273.5 (214.2–344.8)	0.961	268.0 (215.0–355.0)	279.0 (218.0–346.5)	0.916
Preoperative CRP, n (%)						
< 8	43 (19.2%)	57 (24.4%)	0.181	38 (19.9%)	46 (24.1%)	0.323
≥ 8	181 (80.8%)	177 (75.6%)		153 (80.1%)	145 (75.9%)	
Anemia, n (%)						
No	87 (38.8%)	96 (41.0%)	0.633	78 (40.8%)	78 (40.8%)	1.000
Yes	137 (61.2%)	138 (59.0%)		113 (59.2%)	113 (59.2%)	
Preoperative albumin, median (IQR)	31.6 (28.1–35.1)	33.1 (29.4–36.6)	0.006	32.2 (28.8–35.5)	32.6 (28.7–35.4)	0.922
Preoperative creatinine, median (IQR)	81.5 (60.8–108.2)	78.5 (59.0–108.0)	0.678	82.0 (60.0–110.5)	76.0 (59.0–105.0)	0.286
Smoking, n (%)						
No	165 (73.7%)	176 (75.2%)	0.703	140 (73.3%)	143 (74.9%)	0.726
Yes	59 (26.3%)	58 (24.8%)		51 (26.7%)	48 (25.1%)	
Drinking, n (%)						
No	171 (76.3%)	178 (76.1%)	0.946	144 (75.4%)	145 (75.9%)	0.905
Yes	53 (23.7%)	56 (23.9%)		47 (24.6%)	46 (24.1%)	
Hypertension, n (%)						
No	118 (52.7%)	93 (39.7%)	0.006	95 (49.7%)	86 (45.0%)	0.356
Yes	106 (47.3%)	141 (60.3%)		96 (50.3%)	105 (55.0%)	
Kidney disease, n (%)						
No	168 (75.0%)	155 (66.2%)	0.040	139 (72.8%)	136 (71.2%)	0.732
Yes	56 (25.0%)	79 (33.8%)		52 (27.2%)	55 (28.8%)	
LEAD, n (%)						
No	74 (33.0%)	73 (31.2%)	0.673	61 (31.9%)	67 (35.1%)	0.515
Yes	150 (67.0%)	161 (68.8%)		130 (68.1%)	124 (64.9%)	
Preoperative bacterial culture, n (%)						
Negative	83 (37.1%)	103 (44.0%)	0.129	72 (37.7%)	82 (42.9%)	0.297
Positive	141 (62.9%)	131 (56.0%)		119 (62.3%)	109 (57.1%)	
Wagner score, n (%)						
1–3	160 (71.4%)	173 (73.9%)	0.548	140 (73.3%)	144 (75.4%)	0.639
4–5	64 (28.6%)	61 (26.1%)		51 (26.7%)	47 (24.6%)	
Number of toes, n (%)						
1	163 (72.8%)	170 (72.6%)	0.977	137 (71.7%)	140 (73.3%)	0.731
2–5	61 (27.2%)	64 (27.4%)		54 (28.3%)	51 (26.7%)	

*BMI* body mass index, *CRP* C-reactive protein, *IQR* interquartile range, *LEAD* lower extremity arterial disease, *LOS* length of stay, *TIR* time in range

### Clinical outcomes

As shown in Table 2, the two groups were further compared, including re-amputation, postoperative infection, length of stay (LOS), costs, and incision healing on discharge, to assess the difference in outcomes. Among patients with TIR < 70%, 77 patients underwent a second toe amputation within 1 year, and 30 patients died within 1 year. Because of 42 patients who underwent a second operation and 13 patients who died within 1 year, patients in the TIR ≥ 70% group had a better prognosis. The postoperative infection rate was significantly lower in the TIR ≥ 70% group than in the TIR < 70% group (9.95% versus 18.32%, P = 0.019). Besides, compared with the control group, the length of stay in the TIR ≥ 70% group showed significantly shorter (P = 0.032), but there was no statistically difference in the hospitalization costs between the two groups (P = 0.088). At discharge, 51.3% of the wounds in the TIR < 70% group were not fully healed, significantly higher than those in the TIR ≥ 70% group (P < 0.001).

**Table 2:** Postoperative outcomes

Outcomes	TIR < 70% (n = 191)	TIR ≥ 70% (n = 191)	*P*-value
Re-amputation, n (%)	77 (40.3%)	42 (22.0%)	< 0.001
Postoperative infection, n (%)	35 (18.32%)	19 (9.95%)	0.019
LOS, median (IQR)	25.0 (17.0–34.0)	21.0 (15.5–30.0)	0.032
Costs, median (IQR)	50,151.0 (33,420.7–78,547.2)	45,102.0 (33,536.4–64,069.5)	0.088
Incision healing on discharge, n (%)			
Complete healing	93 (48.7%)	139 (72.8%)	< 0.001
Incomplete healing	98 (51.3%)	52 (27.2%)	

*IQR* interquartile range, *LOS* length of stay, *TIR* time in range

### Risk factors for re-amputation

Univariate analysis for risk factors associated with surgery of re-amputation showed that re-amputation was associated with smoking (P = 0.010), lower extremity arterial disease (P < 0.001), and TIR < 70% (P < 0.001). Multivariate analysis revealed that smoking, lower extremity arterial disease and TIR < 70% were risk factors for surgery of re-amputation (Table 3).

**Table 3:** Univariate and Multivariate Analysis of Re-amputation

Variables	Cases without reamputation, (n = 263)	Cases with reamputation, (n = 119)	Univariate analysis *P*	Multivariate analysis *P*
Age, median (IQR), years	67.0 (58.0–74.0)	67.0 (62.0–72.0)	0.658	
Sex, n (%)				
Male	178 (67.7%)	84 (70.6%)	0.571	
Female	85 (32.3%)	35 (29.4%)		
BMI, median (IQR), kg/m^2^	23.1 (21.1–25.4)	22.7 (21.6–25.0)	0.705	
Preoperative blood glucose, n (%)				
< 6.1	54 (20.5%)	28 (23.5%)	0.509	
≥ 6.1	209 (79.5%)	91 (76.5%)		
Preoperative HbA1c, n (%)				
< 7.5	61 (23.2%)	25 (21.0%)	0.636	
≥ 7.5	202 (76.8%)	94 (79.0%)		
Preoperative WBC, median (IQR)	8.7 (6.9–11.6)	9.0 (6.8–12.9)	0.292	
Preoperative neutrophil, median (IQR)	6.4 (4.5–9.1)	6.4 (4.8–10.4)	0.336	
Preoperative lymphocyte, median (IQR)	1.5 (1.1–1.8)	1.5 (1.1–1.8)	0.849	
Preoperative platelet, median (IQR)	268.0 (209.0–353.0)	287.0 (226.5–343.5)	0.303	
Preoperative CRP, n (%)				
< 8	63 (24.0%)	21 (17.6%)	0.168	
≥ 8	200 (76.0%)	98 (82.4%)		
Anemia, n (%)				
No	112 (42.6%)	44 (37.0%)	0.302	
Yes	151 (57.4%)	75 (63.0%)		
Preoperative albumin, median (IQR)	32.9 (29.0–35.7)	31.4 (28.4–35.2)	0.188	
Preoperative creatinine, median (IQR)	78.0 (59.0–108.0)	84.0 (61.5–112.5)	0.372	
Smoking, n (%)				
No	205 (77.9%)	78 (65.5%)	0.010*	0.016*
Yes	58 (22.1%)	41 (34.5%)		
Drinking, n (%)				
No	202 (76.8%)	87 (73.1%)	0.436	
Yes	61 (23.2%)	32 (26.9%)		
Hypertension, n (%)				
No	125 (47.5%)	56 (47.1%)	0.932	
Yes	138 (52.5%)	63 (52.9%)		
Kidney disease, n (%)				
No	194 (73.8%)	81 (68.1%)	0.251	
Yes	69 (26.2%)	38 (31.9%)		
LEAD, n (%)				
No	111 (42.2%)	17 (14.3%)	< 0.001*	< 0.001*
Yes	152 (57.8%)	102 (85.7%)		
Preoperative bacterial culture, n (%)				
Negative	114 (43.3%)	40 (33.6%)	0.073	
Positive	149 (56.7%)	79 (66.4%)		
Wagner score, n (%)				
1–3	200 (76.0%)	84 (70.6%)	0.258	
4–5	63 (24.0%)	35 (29.4%)		
Number of toes, n (%)				
1	187 (71.1%)	90 (75.6%)	0.359	
2–5	76 (28.9%)	29 (24.4%)		
TIR				
< 70%	114 (43.3%)	77 (64.7%)	< 0.001*	< 0.001*
≥ 70%	149 (56.7%)	42 (35.3%)		

*BMI* body mass index, *CRP* C-reactive protein, *IQR* interquartile range, *LEAD* lower extremity arterial disease, *LOS* length of stay, *TIR* time in range

*Statistical difference between the two groups

### Subgroup analysis

**Fig. 2:**
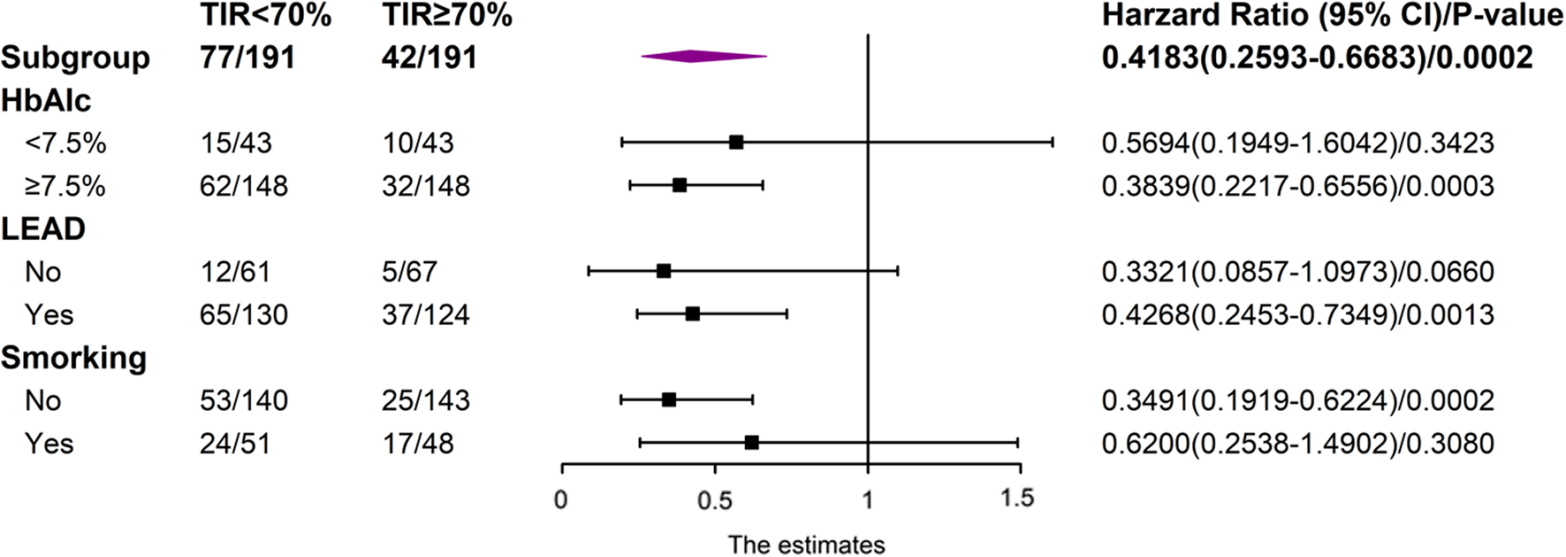
Subgroup analyses of TIR on re-amputation regarding HbA1c, LEAD, and smoking. *CI* confidence interval, *OR* odds ratio, *TIR* time in range, *LEAD* lower extremity arterial disease

Finally, subgroup analyses were performed to explore heterogeneity in our study (Fig. 2). Indicators with statistical differences in Table 3 and HbA1c were selected for further subgroup analysis. The results showed that the TIR < 70% was associated with a greater risk of re-amputation in patients with HbA1c < 7.5%, LEAD, and non-smokers.

## Discussion

Diabetic foot is a global medical and health problem, causing substantial medical and economic burdens to patients and families [21, 22]. Diabetic foot ulcers account for approximately 85% of diabetic patients undergoing non-traumatic lower limb amputations [23]. In addition, more than half of amputees die within 5 years. Therefore, the prevention of amputation is essential to increase the daily quality of life and survival of DFU patients, especially those who have previously undergone amputation surgery [6, 7].

So far, studies on the risk factors of patients undergoing toe amputation have never stopped. In previous studies, the risk factors of patients with DFU are diverse. For example, stabilizing blood glucose has the ability to significantly reduce the risk of amputation in patients with diabetic foot ulcers [9]. After a series of landmark experiments including the Diabetes Control and Complications Trial, HbA1c has become a 'gold standard’ for blood glucose control in clinical practice [24]. In addition, the increase of HbA1c significantly increased the mortality of patients with diabetic foot [25, 26]. However, HbA1c has some limitations as it does not provide information on daily low or high blood sugar or short-term fluctuations. As a single indicator, it only reflects average blood glucose levels over the past three months or so. Studies have found that even patients with roughly the same HbA1c can have wildly different blood sugar fluctuations [27]. However, TIR values can provide indicators including hypoglycemia, hyperglycemia, and glucose fluctuations, and may make up for the defect of HbA1c values [13]. Increasing evidence shows that Time in Range is closely associated with diabetes-related complications [28, 29] and mortality [30, 31] in patients with diabetic foot ulcers.

Compared with HbA1c as a long-term indicator, Time in Range is an intuitive indicator, because it is closely related to the short-term health and disease status of patients. Generally, there are two methods for measuring blood glucose in diabetic patients: CGM data and seven-point blood glucose data. While CGM data is more accurate in blood glucose monitoring than the seven-point test, it also means more expensive and equipment dependent [32]. Despite the continuous use of CGM technology, the Seven-point Blood glucose test using capillary samples is still the primary method for measuring blood glucose levels in most patients with diabetic foot ulcers [33]. Therefore, seven-point blood glucose test was used in this study to obtain TIR data.

As far as we know, we’re the first to report the impact of Time in Range during hospitalization on outcomes in patients with toe amputation. This study was aimed to investigate the more specific and comprehensive effects of TIR on diabetic patients with toe amputation. Before matching, the TIR < 70% group had a higher WBCs count, higher neutrophil counts, higher blood glucose at admission, lower rates of hypertension, lower rates of kidney disease, and lower albumin levels. After matching, the baseline data of 191 patients in each group were balanced and there was no statistical difference. The results of our study showed differences in re-amputation, postoperative infection, length of hospital stay (Los), and wound healing on discharge between the two groups. In addition, our results showed that smoking, lower extremity arterial disease, and TIR < 70% were risk factors for re-amputation.

To some extent, the history of toe amputation reflects the disease progression of diabetic patients. One study found that the risk of a below-knee amputation was increased by a history of minor amputations [34]. In addition, a meta-analysis showed that patients with a history of amputation had a nearly 50 percent higher risk of having a second amputation than those with no history of amputation [35], which was similar to our study results (Table 2). Patients who have had a previous amputation rely only or more on the opposite limb to walk, making previously healthy limbs more vulnerable to overload and recurrent ulcers. For DFU patients who have undergone amputation, corresponding preventive measures should be taken according to risk factors to reduce the occurrence of reoperation. This is why this study focused more on patients with toe amputation than on patients only with diabetic foot.

The above studies indicate that TIR is an important indicator of diabetes and has attracted more and more attention. This study concluded that TIR was negatively correlated with the rate of toe re-amputation, poor wound healing, and postoperative infection in patients with DFU. Unstable blood glucose, especially high blood glucose, can stimulate monocytes to release inflammatory factors, which further aggravate the inflammatory response, and ultimately affect the normal wound healing process [36]. In addition, the continuous presence of high glucose in the wound can lead to the formation and accumulation of glycation end products, leading to delayed wound healing [37]. Repeated inflammatory stimulation and postoperative infection tend to lead to difficult healing of the wound after toe amputation, and even cause osteomyelitis and eventually require a second toe amputation.

Multivariate analysis for risk factors associated with surgery of re-amputation showed that smoking and lower extremity arterial disease were associated with second toe amputation. A study demonstrated the act of smoking is strongly associated with amputation in diabetic foot patients, and the act of quitting smoking is considered an effective measure to prevent amputation [38]. Consistent with our study, studies have proved that smoking can increase the 30-day readmission rate of DFU patients after lower limb amputation [39]. Lower limb arterial disease is also considered a risk factor for amputation in diabetic foot patients. Faglia et al. found that the 5-year rate of amputation was increased in diabetic patients who were diagnosed with lower limb artery disease and did not undergo associated vascular remodeling [40]. Finally, our subgroup analysis showed that among diabetic patients with relatively normal HbA1c, patients with lower TIR and LEAD were at greater risk for future re-toe amputations. This means that the combination of glycated hemoglobin, which represents a long-term glycemic index, and TIR, which fluctuates in the short term, will be more beneficial to clinical practice.

However, there are still several limitations that need to be mentioned in this study. First, the TIR used in this study was extracted by seven-point data. The seven-point glucose test data have inherent limitations and are similar to but not equivalent to the CGM database. Although the seven-point data method has been proved to be an alternative to CGM data, CGM is the most formal method to obtain TIR for monitoring blood glucose in the future. Secondly, this study is a retrospective clinical study. Although propensity matching score we used to reduce differences in baseline data of patients enrolled in the study, the rigor of prospective clinical study is difficult to achieve by other research methods. Finally, this research is single-center clinical research, and further research should be conducted in various places to obtain more reliable results.

## Conclusion

In summary, a lower TIR during a DFUs patient's hospital stay was associated with toe re-amputation in six months, postoperative infection, longer length of stay, and poor wound healing. TIR can be used as a short-term indicator of glycemic control in DFUs patients and should be applied appropriately in clinical practice. Moreover, a future multicenter prospective study is needed to determine the relationship between toe re-amputation and TIR in patients with DFUs.

## Data Availability

The datasets used and/or analyzed during the current study are available from the corresponding author on reasonable request.
